# Correction: Asymmetries in mother-infant behaviour in Barbary macaques (*Macaca sylvanus*)

**DOI:** 10.7717/peerj.4736/correction-1

**Published:** 2018-08-23

**Authors:** Barbara Regaiolli, Caterina Spiezio, William Donald Hopkins

**Affiliations:** 1 Research & Conservation Department, Parco Natura Viva—Garda Zoological Park, Verona, Italy; 2 Neuroscience Institute and Language Research Center, Georgia State University, Atlanta, GA, United States of America

Corrigendum:

After publication, the authors noted that Figures 1 and 2 are not the final versions of the figures. Both figures have now been amended to include: correct representations of the upper and lower quartiles, outliers, minimum and maximum values. In particular, in the new boxplots, the whiskers extend up from the top of the box to the largest data element that is less than or equal to 1.5 times the interquartile range (IQR) and down from the bottom of the box to the smallest data element that is larger than 1.5 times the IQR. Values outside this range are considered to be outliers and are drawn as points. Additionally, each measure has a different pattern and each interval in the Y axis is labelled with extended lines. In Figure 2, the Y axis has been renamed Absolute Laterality Index.

